# Potential of Vasoprotectives to Inhibit Non-Enzymatic Protein Glycation, and Reactive Carbonyl and Oxygen Species Uptake

**DOI:** 10.3390/ijms221810026

**Published:** 2021-09-16

**Authors:** Katarzyna Bednarska, Izabela Fecka

**Affiliations:** Department of Pharmacognosy and Herbal Medicines, Faculty of Pharmacy, Wroclaw Medical University, ul. Borowska 211, 50-556 Wroclaw, Poland; izabela.fecka@umed.wroc.pl

**Keywords:** methylglyoxal trapping, reactive carbonyl species, vasoprotective, antiglycation activity, advanced glycation end products, antioxidant activity, diabetes complications

## Abstract

Reactive carbonyl species (RCS) such as methylglyoxal (MGO) or glyoxal (GO) are the main precursors of the formation of advanced glycation end products (AGEs). AGEs are a major factor in the development of vascular complications in diabetes. Vasoprotectives (VPs) exhibit a wide range of activities beneficial to cardiovascular health. The present study aimed to investigate selected VPs and their structural analogs for their ability to trap MGO/GO, inhibit AGE formation, and evaluate their antioxidant potential. Ultra-high-performance liquid chromatography coupled with an electrospray ionization mass spectrometer (UHPLC-ESI-MS) and diode-array detector (UHPLC-DAD) was used to investigate direct trapping capacity and kinetics of quenching MGO/GO, respectively. Fluorimetric and colorimetric measurements were used to evaluate antiglycation and antioxidant action. All tested substances showed antiglycative effects, but hesperetin was the most effective in RCS scavenging. We demonstrated that rutin, diosmetin, hesperidin, and hesperetin could trap both MGO and GO by forming adducts, whose structures we proposed. MGO-derived AGE formation was inhibited the most by hesperetin, and GO-derived AGEs by diosmetin. High reducing and antiradical activity was confirmed for quercetin, rutin, hesperetin, and calcium dobesilate. Therefore, in addition to other therapeutic applications, some VPs could be potential candidates as antiglycative agents to prevent AGE-related complications of diabetes.

## 1. Introduction

In patients with long-term uncontrolled hyperglycemia in diabetes, pathological structural-functional changes in the vascular endothelium are observed [1]. These are mainly associated with increased non-enzymatic glycation of proteins and consequently with excessive formation and deposition of advanced glycation end products (AGEs) in the extracellular space and within cells of the blood vessel wall [2]. Vascular complications are the main cause of morbidity and mortality in type 2 diabetes mellitus (T2DM) [3]. Currently, there are no effective pharmacological strategies known to prevent vascular endothelial damage in diabetic patients [4]. AGE formation in vivo is mostly attributed to the reaction of carbonyl groups of 1,2-dicarbonyl compounds (reactive carbonyl species, RCS) such as methylglyoxal (MGO) or glyoxal (GO) with the free amino groups of proteins and other biomacromolecules, resulting in the formation of covalently cross-linked aggregates [5]. Chemically, the mechanism of this reaction is based on nucleophilic addition to the carbonyl group, and the resulting products are intermediates that further undergo transformation by various other chemical reactions to the advanced glycation end products [6]. In diabetic patients, the concentration of RCS is elevated by up to 6-fold; it enhances the non-enzymatic process of protein glycation and subsequent formation of AGEs [7]. RCS-mediated AGEs affect the stability of blood vessel walls by reducing their integrity and consequently increasing permeability [8]. They induce local inflammation through the activation of protein kinase C and nuclear factor NF-kB, which leads to increased synthesis and secretion of proinflammatory cytokines and stimulation of macrophages and neutrophils [9]. They also contribute to excessive secretion of prothrombotic factors and decreased sensitivity to vasodilatory agents [10]. However, 1,2-dicarbonyl compounds, particularly methylglyoxal, adversely affect the vascular wall not only indirectly through induction of AGE formation, but also directly [11]. MGO promotes oxidative stress by inducing the formation of hydrogen peroxide (H_2_O_2_), superoxide anion radical (O^2−^), and peroxynitrite anion (ONOO^−^), impairing the antioxidant defense system and reducing the intracellular level of glutathione [12]. Methylglyoxal is also able to induce apoptosis by increasing the Bax/Bcl-2 ratio, activation of caspase-9 and caspase-3, and promoting the mitochondrial apoptosis pathway [13]. The proven participation of RCS and AGEs in the development of endothelial damage has prompted research on the potential use of RCS-trapping, antiglycative, and antioxidant compounds as protective agents in diabetic complications [14]. Among several mechanisms that may potentially reduce the levels of RCS and AGEs in the system, one is direct trapping of 1,2-dicarbonyls, resulting in the formation of specific adducts [15]. This activity has already been proven in several in vitro studies for quercetin [16], catechin [17], epicatechin [18], genistein [19], luteolin, kaempferol, and naringenin [20]. The in vitro studies also revealed that the trapping mechanism occurs under physiological conditions—the formation of adducts of methylglyoxal and myricetin in mice as well as methylglyoxal and metformin in humans has been demonstrated [21,22].

According to the Anatomical Therapeutic Chemical Classification System (ATC code), drugs in the vasoprotective category (C05) can be divided into several groups including antivaricose therapy agents (C05B) such as calcium dobesilate (C05BX01) and capillary stabilizers (C05C), which include bioflavonoids (C05CA) such as rutin (C05CA 01), diosmin (C05CA 03), and troxerutin (C05CA 04). They are most often used in conditions such as hemorrhoids, varicose veins, and poor circulation (venous stasis) [23]. However, many scientific studies over the years have demonstrated the protective, multidirectional effects of flavonoids on the cardiovascular system [24,25]. It has been shown that flavonoids can benefit vascular health through antioxidant activity, inhibiting the reactions leading to the production of reactive oxygen species (ROS) [26]. Moreover, flavonoids show spasmolytic activity by inhibiting cyclic adenosine monophosphate (cAMP), which results in vascular smooth muscle relaxation [27]. The inhibition of proteolytic enzymes exhibited by bioflavonoids leads to the strengthening of connective tissue in the blood vessel wall, increasing its sealing and flexibility [28,29]. Nevertheless, the chemical structure of selected compounds from the C05 category may also suggest their potential trapping activity toward reactive carbonyl compounds.

Despite the therapeutic use of vasoprotective (phlebotropic) medicines over the past few decades, little attention has been paid to the molecular mechanisms underlying their potential protective effects in vascular endothelial damage induced by RCS. Therefore, the aim of this study was to verify whether selected substances used as vasoprotective agents (bioflavonoids C05CA and calcium dobesilate used in the treatment of varicose veins C05BX) and their structural analogs (hesperidin and aglycones, except quercetin not used in therapy) have methylglyoxal and glyoxal trapping potential, capacity to inhibit RCS-induced non-enzymatic protein glycation, and exhibit antioxidant activity. Furthermore, for compounds demonstrating the ability to trap RCS, the reaction of methylglyoxal and glyoxal scavenging was studied over time. The antiglycation and antioxidant effects of phlebotropic substances were compared to analogous properties of metformin, the primary oral antidiabetic drug. The findings may shed new light on the potential prospective use of VPs in preventing vascular endothelial damage caused by reactive carbonyl compounds and non-enzymatic glycation.

## 2. Results and Discussion

### 2.1. Antiglycation Assay in RCS-BSA Model In Vitro

Several studies over the years have documented that the process of non-enzymatic protein glycation contributes to the onset and progression of many chronic diseases including diabetes [30]. A great effort has been dedicated to identifying clinically relevant agents able to inhibit AGE formation to delay or prevent the consequences of the glycation process [31]. Aminoguanidine (AG), which is effective at inhibiting glycation, has been tested in clinical trials to alleviate diabetes-related complications. These trials showed that AG provided some beneficial effect on diabetic complications, but severe side effects ruled it out as a drug candidate [32]. Therefore, the search for compounds that can effectively inhibit glycation is still ongoing.

**Figure 1:**
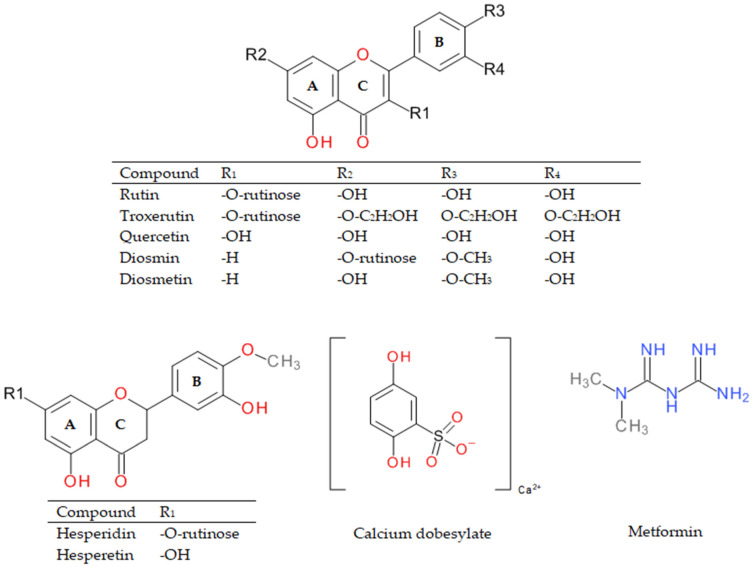
Chemical structures of rutin, quercetin, troxerutin, diosmin, diosmetin, hesperidin, hesperetin, calcium dobesilate, and metformin.

Under in vivo conditions, reactive dicarbonyl compounds are the main inducers of the formation of advanced glycation end products; hence, we decided to investigate compounds with vasoprotective potential and their structural analogs (troxerutin, rutin, quercetin, hesperidin, hesperetin, calcium dobesilate) for RCS-mediated AGE inhibitory activity. Inhibition of their formation was measured using an in vitro biological model where bovine serum albumin (BSA) served as the protein target and methylglyoxal (MGO-BSA-model) or glyoxal (GO-BSA-model) as the glycating agent. The chemical structures of the tested compounds are shown in Figure 1. Aminoguanidine proved to be one of the compounds with the strongest antiglycation activity and was used as a known reference inhibitor of the glycation process. For comparison, metformin used as an antidiabetic agent, whose glycation-inhibiting effect has also been shown in several studies [33,34], was also examined.

**Figure 2:**
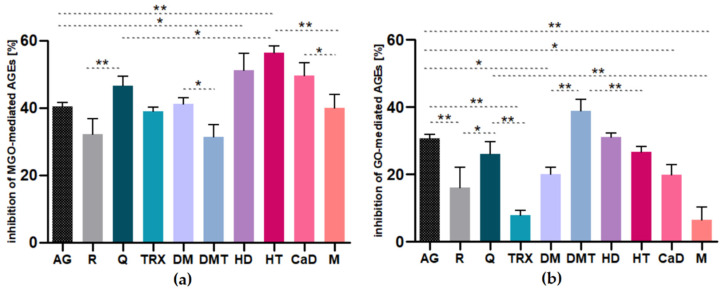
Anti-glycation activity after seven days of incubation of bovine serum albumin with glycative agents (5 mM) and tested compound (1 mM) expressed as % inhibition of: (**a**) MGO-mediated-AGE formation, (**b**) GO-mediated-AGE formation. The results are representative of three experiments performed in triplicate ± SD. Data were analyzed by one-way analysis of variance ANOVA (*p* < 0.0001) followed by Tukey’s multiple comparison test; only significant differences are displayed: * *p* < 0.05, ** *p* < 0.01. Abbreviations: AG, aminoguanidine; R, rutin; Q, quercetin; TRX, troxerutin; DM, diosmin; DMT, diosmetin; HD, hesperidin; HT, hesperetin; CaD, calcium dobesilate; M, metformin.

This study found beneficial effects of all tested VPs and their structural analogs (aglycones) on the inhibition of AGE formation as observed in MGO-BSA and GO-BSA models. Nevertheless, their activity varied. Results expressed as percentage of inhibition of RCS-mediated AGEs are shown in Figure 2.

In the MGO-BSA-model, hesperetin (56.50 ± 1.93%), hesperidin (51.52 ± 0.51%), calcium dobesilate (49.67 ± 4.08%), and quercetin (46.62 ± 1.75%) were found to be the most potent inhibitors of AGE formation. The activity of aminoguanidine used as the reference inhibitor was lower (40.46 ± 1.17%) than for the above-mentioned substances and comparable to the activity of diosmin (41.19 ± 3.78%), metformin (40.00 ± 2.86%), and troxerutin (39.05 ± 1.08%). Activity of the reference inhibitor used was higher only than the activity of rutin (33.46 ± 1.66%) and diosmetin (31.30 ± 5.07%). The results were slightly different for the GO-BSA-model, where diosmetin (38.82 ± 1.08%) and hesperidin (31.13 ± 3.58%) were the most potent inhibitors of glycation, their activity exceeding the activity of aminoguanidine (30.62 ± 1.28%). Hesperetin (26.74 ± 1.93%), quercetin (26.21± 2.00%), rutin (21.33 ± 0.85%), and next calcium dobesilate (19.94 ± 3.89%) and diosmin (19.96 ± 3.51%) showed similar, slightly less potent activity, while the poorest glycation inhibitory capacity was exhibited by troxerutin (7.85 ± 1.41%) and metformin (6.39 ± 1.63%).

In our study, hesperetin and hesperidin showed the most potent antiglycation activity in the model with MGO and a moderately strong effect in the model with GO. A study by Li et al. [35] reported the relatively strong activity of hesperetin (inhibition rate 56.7%) in a BSA model with glucose as the glycating agent and was more potent than its 7-*O*-rutinoside derivative, hesperidin (46.8%). This indicates that the free hydroxyl group at C-7 in ring A contributes to the antiglycative effect. Our observations are in agreement with reports of Matsuda et al. [36] where blocking of the 7-hydroxyl group reduced the antiglycation action. We observed a similar relationship for quercetin and troxerutin (3′,4′,7-tris[*O*-(2-hydroxyethyl)]rutin). In both biological models, the quercetin aglycone exhibited greater AGE inhibitory activity compared with troxerutin. Rutin (quercetin-3-*O*-rutinoside) also inhibited the process of protein glycation to a lesser extent than quercetin. Matsuda et al. [36] suggested that methylation of the hydroxyl group at the C-4′ position of the B ring of flavonoids increases the antiglycative effect. Indeed, in the model with MGO as the glycating agent, hesperetin had the highest activity, and in the GO-BSA-model it was diosmetin; both compounds have a methoxyl group at the C-4′ position. The obtained results also showed that 7-*O*-rutinoside/aglycone pairs like diosmin/diosmetin and hesperidin/hesperetin showed different relationships in both tests. Flavones (with a double bond at C-2/C-3 of the C ring) were characterized by higher aglycone activity in the GO-BSA assay, and flavanones (without a double bond at C-2/C-3 of the C ring) by higher aglycone activity in the MGO-BSA assay. However, this observation requires further explanation. Among quercetin derivatives, the aglycone showed a higher effect than glycosides in both models.

Calcium dobesilate (CaD) is particularly noteworthy among the non-flavonoid compounds we studied. CaD is an angioprotective agent proposed to treat diabetic retinopathy (DR) by protecting against retinal vascular damage [37]. It can slow progression of DR during long-term oral treatment and prevent intravascular and extravascular retinal hemorrhages, reduce the frequency of exudate formation, and enhance visual acuity by reducing microvascular permeability [38]. Our study suggests that CaD also possesses potent activity to inhibit MGO- and GO-induced AGE formation.

In the antiglycation assays, we used aminoguanidine as a reference with well-known glycation inhibitory activity. We also decided to test metformin, a first-line drug for the management of diabetes type 2 because both compounds have a guanidine-derived structure. We found that the glycation inhibitory activity of both compounds in the methylglyoxal model was very similar at about 40%. However, in the model with glyoxal as the glycation agent, we observed that aminoguanidine exceeded the inhibitory activity of metformin by more than 6-fold. In a study by Mehta et al. [39], aminoguanidine and metformin were used as RCS scavengers to investigate the prevention of glyoxal toxicity in isolated rat hepatocytes. The authors observed that at comparable concentrations, metformin failed to prevent glyoxal-induced protein carbonylation, whereas aminoguanidine reduced the carbonylation of proteins. These observations may support, at least partially, our findings on the poor effects of metformin on inhibiting non-enzymatic glycation induced by glyoxal.

The process of non-enzymatic protein glycation is tightly linked to the enhanced production of free radicals and non-enzymatic glucose oxidation [37]. The formation of advanced glycation end products is a major source of reactive oxygen species, and the oxidative microenvironment triggered by the accumulation of AGEs can also promote their enhanced production [38]. Therefore, compounds inhibiting the formation of AGEs may act not only through direct quenching of RCS, but also through antioxidant or metal ion chelating activity [39,40]. The results of our experiment indicate that, indeed, in vitro antiglycative action is not only associated with RCS trapping activity. Compounds such as calcium dobesilate, diosmin, and troxerutin lacking the ability to trap methylglyoxal and glyoxal nevertheless exhibited antiglycation activity through other mechanisms. A more in-depth investigation of the relationship between structure and activity, with both quantitative and mechanistic aspects, is necessary to explain and fully understand the experimental observations.

### 2.2. Non-Enzymatic Antioxidant Activity

It is well known that oxidative stress can lead to cell and tissue damage, contributing to vascular endothelial dysfunction. Oxidative stress is also known to play a primary role in methylglyoxal-induced endothelial damage, and it is closely linked to the process of protein glycation [40]. Since restriction of the production of free radicals in the glycation process can decrease the formation of AGEs [41], we decided to investigate VPs and their structural analogs for antioxidant-reducing and antiradical activity.

ABTS (2,2′-azino-bis(3-ethylbenz-thiazoline-6-sulfonic acid assay) and FRAP (ferric reducing antioxidant power assay) are frequently used methods to assess the antioxidant capacity of a biological material, pure compound, or mixture of substances. Both are spectrophotometric techniques based on a single electron transfer mechanism (SET) [42,43]. FRAP allows one to directly determine the reducing ability of the sample [44]. The use of the ABTS assay enables measurement of the total antioxidant activity of the samples [45].

Vasoprotective substances and their structural analogs were tested for antioxidant activity using FRAP and ABTS assays. Metformin was examined to determine whether generally the first-line medication prescribed for type 2 diabetes has a reducing or antiradical activity. Trolox was used to plot the calibration curve in the ABTS test, and iron(II) sulfate solution was used in the FRAP test. Table 1 summarizes the antioxidant activity values expressed as percent inhibition and concentration required for a 50% reduction in radical activity (IC_50_, µM) in the ABTS assay and as an Fe^2+^ iron ion equivalent (Fe(II), µM) in the FRAP assay. In order to clearly show the differences in the activity of the studied compounds in scavenging free radicals and reducing Fe^3+^ ions, we chose the results obtained for concentrations of 4.6 μM and 9.1 μM, respectively.

**Figure 3:**
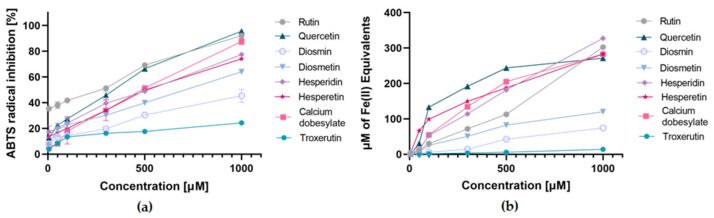
Concentration-dependent antioxidant activity of investigated vasoprotectives in (**a**) ABTS assay expressed as percent of radical inhibition; (**b**) FRAP assay expressed as Fe^2+^ iron ion equivalent (Fe(II) µM); all measurements were performed in triplicate in the concentration range of 5–1000 µM of tested compounds; results are presented as means ± SD (*n* = 3).

The current in vitro assays demonstrated the concentration-dependent antioxidant activity of the tested compounds, as shown in Figure 3. The higher the concentration of the sample used, the analogously greater the antiradical activity. In the FRAP assay, the most potent reducing ability was found for quercetin (133.3 μM Fe^2+)^ and hesperetin (99.3 μM Fe^2+^), followed by calcium dobesilate (55.0 μM Fe^2+^), hesperidin (53.1 Fe^2+^), rutin (30.1 μM Fe^2+^), and diosmetin (25.6 μM Fe^2+^). In contrast, the lowest activity was recorded for diosmin (5.8 μM Fe^2+^) and troxerutin (1.7 μM Fe^2+^), while metformin showed no reducing action.

The ABTS assay showed the greatest antioxidant potential expressed as % inhibition for rutin (69.2%) and quercetin (66.4%), followed by similar inhibitory activity for calcium dobesilate (51.4%), hesperetin (49.8%), and hesperidin (48.9%). Slightly lower inhibition percentage values were observed for diosmetin (37.5%), diosmin (27.6%), and Trolox (29.0%) used as a positive control. The lowest inhibition activity was noted for troxerutin (10.8%), while metformin showed no scavenging activity in this assay. The IC_50_ values for the different tested compounds showed a similar trend as the percent inhibition values. The lowest IC_50_, and consequently the highest antioxidant activity, was demonstrated by rutin (2.4 μM), followed by quercetin (3.8 μM), calcium dobesilate (5.1 μM), hesperidin (5.2 μM), hesperetin (5.7 μM), and diosmetin (7.1 μM). Slightly higher IC_50_ values and thus lower antioxidant activity were shown by diosmin (11.1 μM) and Trolox (11.3 μM), and the lowest by troxerutin (24.1 μM).

The FRAP assay indicated that the reducing activity of aglycones was higher than that of rutinosides—the effect of quercetin/diosmetin/hesperetin was more potent than troxerutin and rutin/diosmin/hesperidin. This pattern has been previously reported, for example, for kaempferol glycosides exhibiting about 30–40% lower antioxidant activity than the kaempferol aglycone [46]. Our study also indicated that glycosylation or ethylation of hydroxyl groups, exemplified by quercetin derivatives, significantly decreased the reducing potential of the compound (quercetin >>> rutin > troxerutin). Blocking the flavonoid phenolic group at C-4’ also diminished the reducing properties (quercetin > hesperetin > diosmetin). In contrast, saturation of the double bond at C-2/C-3 (flavanones vs. flavones) increased activity (hesperidin >>> diosmin, hesperetin >>> diosmetin). These relationships showed that the reducing ability depends mainly on the number of free hydroxyl groups in the molecule and the degree of oxidation of the three-carbon linker in the C-ring. A similar clear structure–activity relationship was not demonstrated using the ABTS test. In both antioxidant assays and the MGO-BSA antiglycation test, hesperidin and calcium dobesilate showed comparable effects.

Interestingly, metformin showed no antioxidant activity in either the FRAP assay or the ABTS assay, however, there are reports that it has the ability to inhibit intracellular formation of ROS [47]. A study by Logie et al. [48] showed that metformin can chelate metal ions, which may be directly responsible for its antioxidant effect. A similar conclusion emerges for troxerutin, which showed very low activity in both antioxidant assays, but some publications have reported its antiradical activity in vivo [49]. This effect can also be explained by chelating activity, confirmed in several studies [50].

It should be noted that high in vitro activity does not necessarily reflect in vivo activity [51]. An important limitation is the bioavailability of vasoprotective flavonoids. Rutinosides are known to have low bioavailability due to their poor solubility and lipophilicity [52]. Although quercetin and hesperetin have shown high reducing and antiradical activity, it should be taken into account that their bioavailability is around 20%; only this amount of orally administered dose reaches the bloodstream [53]. Quercetin taken at 500 mg reaches a plasma concentration of about 1.4 μM [54], and hesperetin (500 mg) about 2.7 μM [55], so they were lower than the IC_50_ concentrations for which the activities were specified in our study. In comparison, calcium dobesilate, whose radical inhibitory capacity was comparable to that of hesperetin (at ~5 μM), reached a plasma concentration of about 19 μM after 500 mg administration [56].

### 2.3. Direct Methylglyoxal/Glyoxal Trapping Capacity

RCS trapping is one of the mechanisms that potentially reduce plasma concentration of methylglyoxal and glyoxal [57]. Therefore, selected VPs and their structural analogs have been examined for their ability to trap reactive carbonyl species. After 1 h incubation of each test compound (rutin, troxerutin, diosmin, diosmetin, hesperidin, hesperetin, calcium dobesilate) with methylglyoxal or glyoxal under simulated physiological conditions, the reaction mixture was further analyzed by UHPLC-MS to detect potentially formed adducts of the test compound with MGO/GO. In the study, quercetin and metformin were used as reference compounds with recognized activity for direct trapping of 1,2-dicarbonyls. The extract ion chromatogram (EIC) mode was used to search for pseudomolecular ions increased by 72 Da/144 Da for mono-MGO/di-MGO adducts or by 58 Da/116 Da for mono-GO/di-GO adducts. All compounds except metformin, for which the positive ion mode was used, were analyzed in negative electrospray ionization mode.

The study revealed that under the experiment conditions quercetin, rutin, diosmetin, hesperetin, hesperidin, and metformin showed activity toward direct trapping of methylglyoxal, whereas glyoxal was directly trapped only by rutin, diosmetin, hesperetin, and hesperidin. The products of the reaction between MGO and GO with tested compounds and the fragmentation pattern of each product are listed in Table 2 and Table 3, respectively. Troxerutin and calcium dobesilate did not trap RCS, probably due to chemical structure differences.

**Figure 4:**
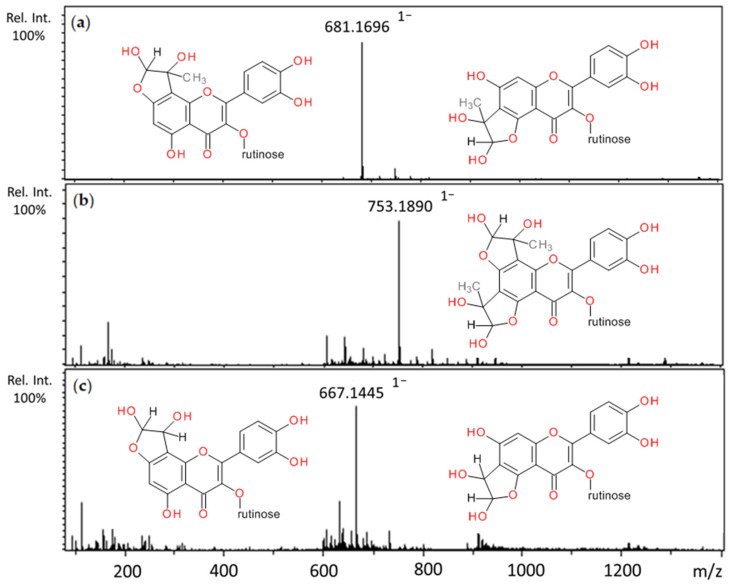
Mass spectra of methylglyoxal and glyoxal adducts with rutin after 1 h incubation in pH 7.4 phosphate buffer solution at 37 °C and their proposed chemical structure: (**a**) mono-MGO-rutin; (**b**) di-MGO-rutin; (**c**) mono-GO-rutin; other isomeric forms are also possible.

In the reaction of rutin with methylglyoxal, after one hour of incubation, the appearance of three major peaks with pseudomolecular ion masses at *m*/*z* 681 and retention times of 8.69, 8.84, 8.85 min, respectively, were observed (Table 2). They corresponded to the molecular weight of mono-MGO-rutin adduct (M*r* 682 Da). All of them produced fragment ions at *m*/*z* 663, indicating elimination (neutral lost) of a water molecule [M–18–H]^−^ and at 609, which suggests loss of one MGO molecule [M–72–H]^−^. Three other peaks a–c with *m*/*z* 753 (Rt 7.90, 8.26, 8.44 min) were also present, and were identified as di-MGO-rutin adducts as they gave fragments at *m*/*z* 681, representing the mass of the di-MGO adduct minus one methylglyoxal moiety [M–72–H]^−^, and 609, indicating the loss of two methylglyoxal molecules [M–144–H]^−^. With glyoxal, rutin formed only mono-GO adducts. Two peaks appeared on the chromatogram with pseudomolecular ions of 667 characterized by retention times of 8.09 and 8.41 min, and since their masses were greater than that of rutin by the mass of a single glyoxal molecule, they were further characterized as mono-GO-rutin adducts (Table 3). The ability of rutin to trap methylglyoxal has been previously reported in several studies [58,59]. Mass spectra of the adducts formed by rutin and MGO/GO and proposed chemical structures are shown in Figure 4.

**Figure 5:**
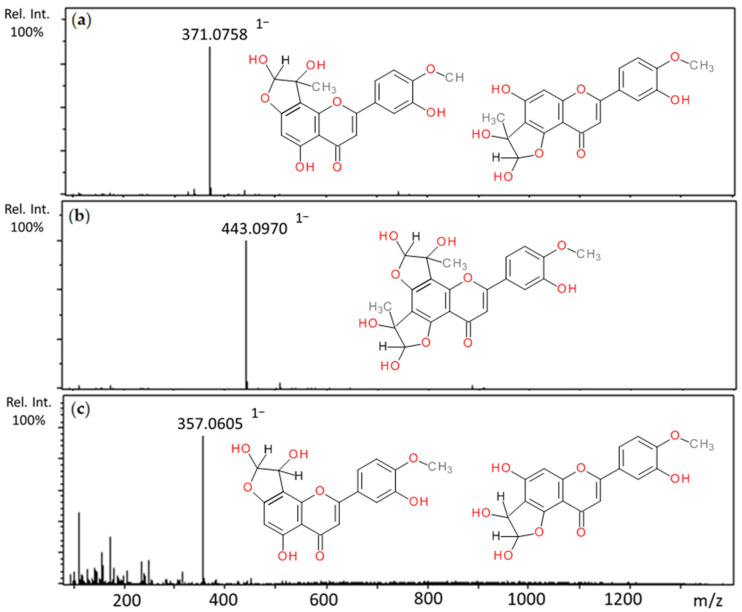
Mass spectra of methylglyoxal and glyoxal adducts with diosmetin after 1 h incubation in pH 7.4 phosphate buffer solution at 37 °C and their proposed chemical structure: (**a**) mono-MGO-diosmetin; (**b**) di-MGO-diosmetin; (**c**) mono-GO-diosmetin; other isomeric forms are also possible.

Incubation of diosmetin with MGO resulted in three chromatographic peaks at 11.24, 12.09, and 12.36 min with *m*/*z* values of 443 and 371 (a and b, Table 2). The first was described as di-MGO-diosmetin because the pseudomolecular ion mass corresponding to diosmetin increased by two methylglyoxal molecules (144 Da) and MS/MS analysis confirmed this—a fragment ion with *m*/*z* 299 corresponding to diosmetin was noted. The other two peaks with *m*/*z* at 371 were 72 Da greater than the pseudomolecular ion of diosmetin; therefore, they were proposed as mono-MGO-diosmetin adducts. The fragmentation pattern indicated that di-MGO-diosmetin and mono-MGO-diosmetin lost a water molecule to form daughter ions at *m*/*z* 425 and 353 [M–18–H]^−^, followed by a methyl group (15 Da) in the B ring resulting in ions at *m*/*z* 410 and 338 [M–18–15–H]^−^, respectively. The reaction of diosmetin and glyoxal led to the formation of two main peaks in the UHPLC chromatogram with pseudomolecular ions at *m*/*z* 357 (Rt 11.64 and 12.10 min), both heavier by 58 Da than the pseudomolecular ion of diosmetin (*m*/*z* 299), and therefore were assigned as mono-GO-diosmetin adducts (Table 3). The fragment ions observed in the MS/MS analysis similarly showed that both mono-MGO-diosmetin and mono-GO-diosmetin first lost the water and then the methyl group [M–18–15–H]^−^. Mass spectra of the adducts formed by diosmetin and MGO/GO and the proposed chemical structures are shown in Figure 5.

**Figure 6:**
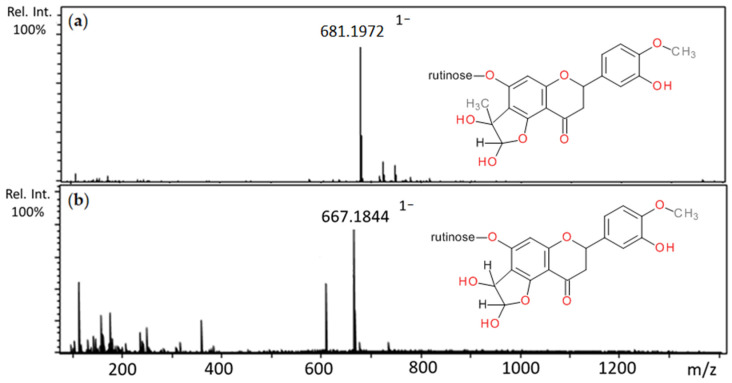
Mass spectra of methylglyoxal and glyoxal adducts with hesperidin after 1 h incubation in pH 7.4 phosphate buffer solution at 37 °C and their proposed chemical structure: (**a**) mono-MGO-hesperidin; (**b**) mono-GO-hesperidin; other isomeric forms are also possible.

Hesperidin and methylglyoxal after 1 h of incubation produced six chromatographic peaks with the pseudomolecular ions at *m*/z 681, and retention times 8.97, 9.19, 9.82, 9.95, 10.88, and 10.98 min (Table 2). The fragmentation pattern was the same for each ion; three principal daughter ions were produced with masses at *m*/*z* 609, 373, and 301. The molecular weight of the resulting ions (682 Da) was greater than the molecular weight of hesperidin by 72 Da, and were therefore identified as mono-MGO-hesperidin adducts. The presence of fragment ions at *m*/*z* 609 (the hesperidin pseudomolecular ion) further confirmed this. The fragmentations with *m*/*z* 373 and 301 suggest the loss of the rutinose moiety by mono-MGO-hesperidin [M–308–H]^−^ and methylglyoxal [M–308–72–H]^−^, respectively. After incubation of hesperidin with glyoxal, two major peaks appeared on the chromatogram at 11.05 and 11.37 min with *m*/*z* at 677 (Table 3). Both were recognized as mono-GO-hesperidin adducts as their pseudomolecular ion values were greater than the ion for hesperidin (*m*/*z* 609) by 58 Da. This was later supported by MS/MS data where a daughter ion was observed with the *m*/*z* value of 609 [M–58–H]^−^. A 301 ion was also observed, suggesting, as described above, the loss of rutinose by the hesperidin molecule [609–308–H]^−^. Mass spectra of the adducts formed by hesperidin and MGO/GO and proposed chemical structures are shown in Figure 6.

**Figure 7:**
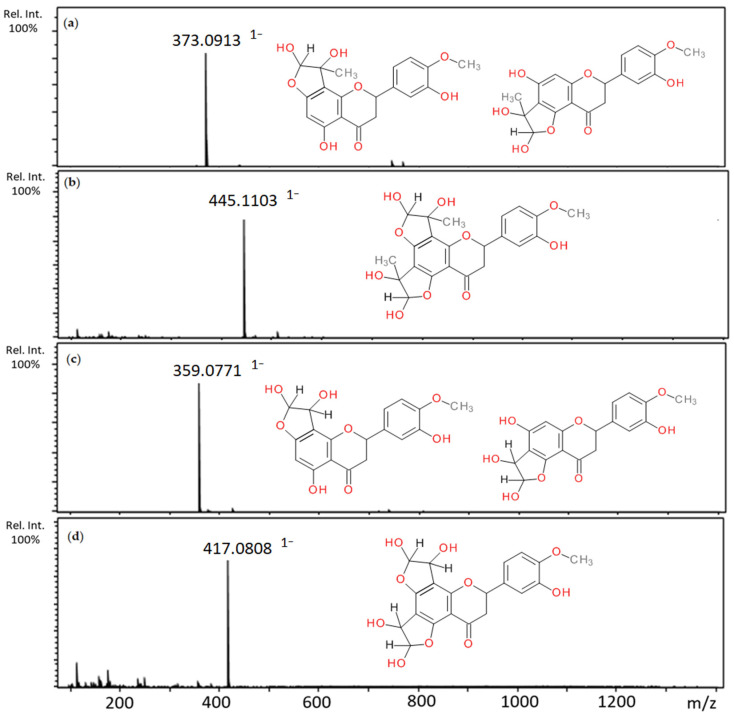
Mass spectra of methylglyoxal and glyoxal adducts with hesperetin after 1 h incubation in pH 7.4 phosphate buffer solution at 37 °C and their proposed chemical structure: (**a**) mono-MGO-hesperetin; (**b**) di-MGO-hesperetin; (**c**) mono-GO-hesperetin; (**d**) di-GO-hesperetin; other isomeric forms are also possible.

After one-hour incubation of hesperetin and methylglyoxal, three major peaks were detected with pseudomolecular ion masses and retention times, respectively: 373 (12.04 and 12.93 min) and 445 (10.80 min). Peaks with an anion at 371, greater than the ion of hesperetin (*m*/*z* 301) by 72 Da, corresponding to the coupling of one molecule of methylglyoxal, were identified as mono-MGO-hesperetin adducts (Table 2). The loss of one water molecule resulted in the most abundant fragment ion of *m*/*z* 355 [M–18–H]^−^. The peak at *m*/*z* 445 was labeled as di-MGO-hesperetin as its molecular weight equaled the mass of the hesperetin plus two MGO molecules (M*r* 446 Da). Similarly, loss of one molecule of water by the di-MGO-hesperetin adduct led to the formation of the most abundant daughter ion with *m*/*z* 427 [M–18–H]^−^. The elimination of the second water molecule resulted in the ion at *m*/*z* 409. Four major peaks with molecular ions at 359 (a–c) and 417 with retention times of 11.28, 12.37, 13.78, 12.99 were observed after 1 h incubation of hesperetin and glyoxal (Table 3). Peaks with *m*/*z* at 359 having pseudomolecular ions heavier than the hesperetin ion (*m*/*z* 301) by 58 Da were considered as mono-GO-hesperetin adducts, as confirmed by MS/MS data. A peak with *m*/*z* 417 greater than the ion for hesperetin by 116 Da (mass corresponding to two glyoxal molecules) was analogously identified as a di-GO-hesperetin adduct. Its fragment ion at 409, reduced by 18 Da (after water loss), was noted on the MS/MS spectrum. Previous studies have demonstrated the ability of hesperetin to form similar adducts with acrolein [60]. Mass spectra of the adducts formed by hesperetin and MGO/GO and the proposed chemical structures are shown in Figure 7.

**Figure 8:**
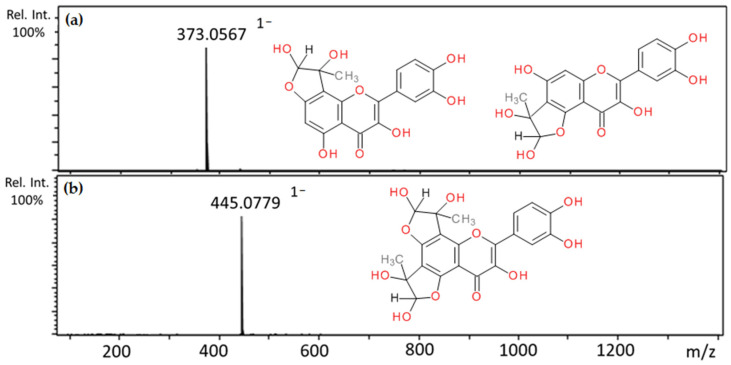
Mass spectra of methylglyoxal adducts with quercetin after 1 h incubation in pH 7.4 phosphate buffer solution at 37 °C and their proposed chemical structure: (**a**) mono-MGO-quercetin; (**b**) di-MGO-quercetin; other isomeric forms are also possible.

Quercetin incubated with methylglyoxal resulted in two peaks characterized as mono-MGO-quercetin and one as di-MGO-quercetin; their pseudomolecular ions were at *m*/*z* 373 (a–b) for the mono-adducts (quercetin precursor ion *m*/*z* 301 increased by 72 Da) and 445 (quercetin precursor ion *m*/*z* 301 increased by 144 Da) for the di-adduct (Table 2). Li et al. [16] reported that quercetin has the potential to trap glyoxal; however, this activity was not observed under the conditions of our study. Mass spectra of the adducts formed by quercetin and MGO and the proposed chemical structures are shown in Figure 8.

**Figure 9:**
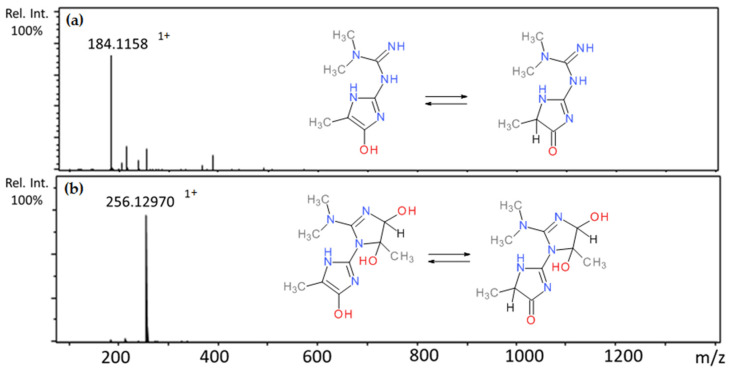
Mass spectra of methylglyoxal adducts with metformin after 1 h incubation in pH 7.4 phosphate buffer solution at 37 °C and their proposed chemical structure (isomers): (**a**) mono-MGO-metformin; (**b**) di-MGO-metformin; other isomeric forms are also possible.

Incubation of metformin and methylglyoxal led to four chromatographic peaks with pseudomolecular ions [M+H]^+^ with 202 at Rt 1.20 min, 184 at 1.46 min, 256 at 1.57 min, and 1.67 min (Table 2). The first ion was described as mono-MGO-metformin due to the ion mass corresponding to metformin increased by one methylglyoxal molecule (72 Da). Based on the literature reports on structural studies of metformin and methylglyoxal adducts [22], the second peak at *m*/*z* 184 was identified as (E)-1,1-dimethyl-2-(5-methyl-4-oxo-4,5-dihy-dro-1H-imidazol-2-yl)guanidine (metformin-MG imidazolinone) and was formed by eliminating one water molecule from mono-MGO-metformin (M*r*, 129 + 72 – 18 = 183 Da). The other two peaks with the ions at *m*/*z* about 256 were attributed to metformin conjugated with two methylglyoxal molecules as their mass, corresponding to the pseudomolecular ion of metformin-MG imidazolinone (*m*/*z* 184), increased by another 72 Da (184 + 72 = 256 Da). The peak with the ion at 184 was dominant compared to the peak with the ion at 202, which may suggest that the monoadduct of metformin with methylglyoxal is more stable after the elimination of one water molecule. However, after 1 h of incubation, both chemical structures of the metformin mono-adduct formed by the reaction were observed. Metformin and glyoxal did not form adducts under conditions of the experiment. Mass spectra of the adducts formed by metformin and MGO and the proposed chemical structures are shown in Figure 9.

### 2.4. Time Course Study of RCS Scavenging Reaction

For compounds demonstrating the ability to directly trap methylglyoxal (rutin, quercetin, diosmetin, hesperidin, hesperetin, metformin) and glyoxal (rutin, diosmetin, hesperidin, hesperetin), a time course study of the trapping reaction was performed. To determine the amount of MGO/GO remaining after incubation at each time point, samples were derivatized with PDA. This method of measuring RCS concentration in various samples is often used in both in vitro and in vivo studies [61].

**Figure 10:**
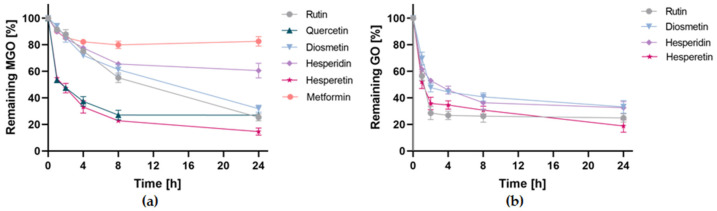
RCS trapping efficiency of tested compounds in pH 7.4 phosphate buffer solution at 37 °C for 1, 2, 4, 8, and 24 h expressed as % of remaining MGO/GO; (**a**) reaction with methylglyoxal; (**b**) reaction with glyoxal. All data are presented as means ± SD (*n* = 2).

Activity over time expressed as a percentage of remaining MGO/GO after reacting with the tested compounds for 1, 2, 4, 8, and 24 h is shown in Figure 10. Data points represent the mean % of remaining MGO/GO with the standard deviation from two independent experiments.

The study showed that methylglyoxal scavenging activity varied among the tested compounds, while glyoxal was scavenged by all tested compounds with quite similar efficacy. Within 24 h, hesperetin, rutin, quercetin, and diosmetin succeeded in trapping 85.41 ± 2.71%, 74.67 ± 2.69%, 73.01 ± 1.89%, and 68.17 ± 2.71% of MGO, respectively, and were found to be the most effective of the tested compounds. At the same time, hesperidin (39.48 ± 5.47%) and metformin (17.42 ± 3.57%) showed the lowest efficiency. Among the most potent compounds, differences in the kinetics of the MGO trapping reaction were evident. Hesperetin and quercetin trapped 50% of methylglyoxal in as little as two hours. In contrast, diosmetin and rutin were only able to trap less than 15% of the MGO at the same time, and both compounds took more than 8 h to quench 50% of the methylglyoxal. Metformin is used in diabetes, for which the ability to trap methylglyoxal was demonstrated in vitro and in vivo. However, the results of our study suggest that its activity in trapping MGO compared to flavonoid substances was relatively low.

After 24 h of incubation, hesperetin and rutin were found to be the most effective in trapping glyoxal by quenching 81.17 ± 4.66% and 75.12 ± 3.11% of GO, respectively. Only slightly less effective was hesperidin with 67.44 ± 4.64% and diosmetin with 66.76 ± 4.76%. Glyoxal seems to be trapped more rapidly in comparison to methylglyoxal. Both hesperetin and rutin were able to quench nearly 50% of glyoxal in just one hour, and diosmetin and hesperidin within 2 h.

The results indicated that among the tested compounds, hesperetin was the most efficient in trapping both methylglyoxal and glyoxal. The weak activity of hesperidin (hesperetin-7-*O*-rutinoside) compared to hesperetin is probably due to the substitution of the hydroxyl group at C-7, resulting in changes in charge distribution in the molecule and therefore decrease general trapping activity [62]. Despite a very similar structure, the quenching ability of MGO and GO was higher for hesperetin than for diosmetin, which is probably attributed to the presence of a double bond at C-2 and C-3 in the heterocyclic ring C of diosmetin. Its presence has been reported to decrease the trapping activity of RCS [63], which is in agreement with our results. The B ring is not thought to play a prominent role in trapping activity. The same study reported that the number of hydroxyl groups in the B ring does not significantly affect the trapping effect [63]. However, the findings of our study suggested that the presence of a substituted hydroxyl group in the B ring at C-4′ stabilizes the molecule, prevents quinone formation, and further C-ring cleavage, thereby preserving the original structure and the consequent trapping activity. This structural feature has also been shown to favorably affect antiglycation potential, possibly via enhancing the RCS trapping activity.

Although our results suggest that selected vasoprotective flavonoids have the ability to trap both methylglyoxal and glyoxal, previous studies have indicated that methylglyoxal is preferred in the trapping reaction. An in vitro study by Li et al. [16] in a model simulating physiological conditions showed that quercetin reduced methylglyoxal concentrations more than twice as efficiently as glyoxal when both compounds were present in solution at the same initial concentration. An in vivo study in humans, on the other hand, showed that although quercetin significantly reduced MGO plasma concentrations in patients (~10%), it had no statistically significant effect on glyoxal concentrations [64]. It should also be noted that according to some studies, compounds sharing the same trapping mechanism can act synergistically; the study of Shao et al. [62] showed that a mixture of quercetin and phloretin traps methylglyoxal more effectively than each compound alone.

The efficiency of RCS trapping in plasma by the investigated substances under in vivo conditions may vary greatly due to the metabolism. Most substances during phase II reactions are conjugated with, for example, glucuronic acid, sulfuric acid, or amino acids [65]. The form to which the drug is metabolized is individual for a given substance and may depend on many factors including the composition of the gut microbiota if absorption occurs in the large intestine (for rutinosides, e.g., hesperidin, diosmin, rutin, troxerutin) [66]. During the phase II reaction, any hydroxyl group of flavonoids can be substituted including those responsible for the trapping activity in ring A (C-5 and C-7), which is likely to reduce the activity of these compounds in plasma [67]. A series of studies related to metabolism is required to assess the exact trapping activity of individual compounds in humans. However, in vitro studies provide the background for clinical trials and are crucial for the selection of potentially useful molecules.

In summary, quercetin, rutin, diosmetin, hesperetin, hesperidin, and metformin were shown to be able to directly trap methylglyoxal, and rutin, diosmetin, hesperetin and hesperidin were shown to trap glyoxal. Hesperetin was found to be the most effective among VPs tested in trapping both methylglyoxal and glyoxal. After 24 h, it scavenged the highest percentage of both RCS.

Additionally, to the best of our knowledge, this is the first time direct RCS trapping activity has been confirmed for hesperetin, hesperidin, and diosmetin. Our study also confirmed the following structure and flavonoid activity relationships: (a) substitution of the hydroxyl group at the C-7 position results in a decrease in the trapping of methylglyoxal and glyoxal, as well as reducing activity; and (b) the double bond in the ring C decreases the MGO/GO trapping and reducing ability. Furthermore, we put forward our hypothesis that the B ring is essential in the trapping activity of flavonoids, which is contrary to earlier reports. Zhu et al. [63] previously claimed that it does not play a significant role in this activity. The findings of our study suggest that the substitution of a phenol group at the C-4’ position of the phenyl ring B stabilizes the molecule, preventing the formation of semiquinone and quinine methide structures, and further cleavage of the heterocyclic C ring. Thus, the original flavonoid structure is preserved and the trapping activity is maintained.

All of the investigated compounds inhibited the formation of RCS-induced AGEs, most likely through several different mechanisms. We observed that the capacity to directly trap RCS was not the only determinant of a potent antiglycative effect, since the ability to inhibit the non-enzymatic process of protein glycation is also greatly influenced by reducing and antiradical (antioxidant) activity. Calcium dobesilate as a potent antioxidant without RCS trapping action showed high antiglycation potential in the MGO-BSA model. In addition, it is characterized by high bioavailability compared to other tested phlebotropic compounds. Among the investigated chemicals with RCS trapping potential, hesperetin was the most potent antiglycative agent in the MGO-BSA model, and in the GO-BSA model, it was shown to be diosmetin.

The antioxidant activity assays showed that the compounds with high reducing and antiradical potential were quercetin, rutin, hesperetin, and calcium dobesilate, and their activities were concentration dependent. It was also confirmed that the activity of flavonoid aglycones was higher than that of glycosides.

## 3. Methods

### 3.1. Chemicals and Standards

Methylglyoxal (40% in water), glyoxal (40% in water), 2,2-diphenyl-1-picrylhydrazyl,2,2-azino-bis-(3-ethylbenzothiazoline-6-sulfonic acid), methanol (HPLC grade), acetonitrile (HPLC gradient grade and LC-MS grade), water (LC-MS grade), diosmin, diosmetin, hesperidin, hesperetin, Trolox, metformin hydrochloride, bovine serum albumin, DMSO, 98–100% formic acid, 2-methylquinoxaline, quinoxaline, o-phenylenediamine, 2, 4, 6-tripyridyl-*s*-triazine, iron(III) chloride hexahydrate, and iron(II) sulfate heptahydrate were purchased from Merck-Sigma-Aldrich (Sigma-Aldrich Sp. z o.o., Poznań, Poland); NaCl, KCl, Na_2_HPO_4_, and KH_2_PO_4_ (reagent grade) were obtained from Chempur (Piekary Śląskie, Poland); quercetin and rutin were from Extrasynthese (Genay Cedex, France); calcium dobesilate was purchased from PPF Hasco-Lek S. A. (Wroclaw, Poland). Water used in the study was glass-distilled and deionized. The stock solutions of standards (3 mM) were prepared by dissolving the appropriate amount of a reference compound in 5 mL of methanol or DMSO. Working standard solutions for the derivatization experiment in the range of 5–210 μM were made by mixing with 50% aq. (aqueous) methanol (*v*/*v*), filtered through hydrophilic Millex Syringe Filters (Durapore 0.22 μm; Millipore, Burlington, MA, USA) and stored at −20 °C.

### 3.2. Antiglycation Assay in BSA-RCS In Vitro Model

The formation of advanced glycation end products was measured following a slightly modified method proposed by Liu et al. [68]. In brief, 90 μM bovine serum albumin was incubated with methylglyoxal and glyoxal at 5 mM in sodium phosphate buffer at 100 mM and pH 7.4, with 0.02% sodium azide (to prevent microbial growth). The compounds investigated for inhibition of non-enzymatic glycation were added at a final concentration of 1 mM. Then, the reaction solution was incubated at 37 °C, shaken at 40 revolutions per minute for seven days in closed vials away from light. Measurement of the fluorescent intensity of total AGEs after incubation was carried out using a Cary Eclipse 500 spectrophotometer (Agilent, Santa Clara, CA, USA) at a wavelength of 350 nm for excitation and 450 nm for emission. Data acquisition was obtained with the Cary Eclipse Control Software (Agilent, Santa Clara, CA, USA). The measurements from three experiments were all performed in triplicate, and the percent inhibition of AGE formation was calculated using the following equation:

Inhibition of RCS-mediated AGES [%] = {1 − [(FI_1_)/(FI_0_)]} × 100

where FI_0_ is mean fluorescence intensity of the blank sample and FI_1_ is the mean fluorescence intensity of the sample.

### 3.3. Antioxidant Activity

#### 3.3.1. ABTS Assay

The ABTS radical scavenging activity was determined according to the slightly modified method described by Chen and Kang [69]. The ABTS^•+^ stock solution was prepared by mixing equal quantities of aqueous solutions of 2,2-diphenyl-1-picrylhydrazyl,2,2-azino-bis-(3-ethylbenzothiazoline-6-sulfonic acid (ABTS, 7.0 mM) and potassium persulfate (K_2_S_2_O_8_, 2.45 mM) and incubating the mixture in the dark at 25 °C for 12 h. The stock solution of ABTS and K_2_S_2_O_8_ was then diluted with methanol to reach absorbance at 734 nm. In a 96-well microplate, 200 µL of ABTS^•+^ reagent was mixed with 20 µL of each tested sample at different concentrations. The plate was incubated for 15 min in the dark at ambient temperature and the absorbance was taken at 517 nm using a Multiskan GO microplate spectrophotometer (Thermo Fisher Scientific; Waltham, MA, USA). All measurements were performed in triplicate using the concentration range of 5–1000 µM of the tested compounds. The standard curve was prepared using different concentrations of Trolox in the range of 100–1000 μM. The ABTS radical scavenging activity was calculated as follows:

ABTS radical inhibition [%] = (A_0_ − A_1_)/A_0_ × 100

where A_0_ is the mean absorbance of the control and A_1_ is the mean absorbance of the sample with ABTS. The IC_50_ values were calculated using linear regression analysis and used to express the antioxidant capacity.

#### 3.3.2. FRAP Assay

The ferric reducing antioxidant power assay (FRAP) was performed according to the method proposed by Benzie and Strain [70] with slight modification. The stock solution of FRAP reagent was obtained by mixing 10 mM 2,4,6-tripyridyl-*s*-triazine (TPTZ) in 40 mM hydrochloric acid with 20 mM iron(III) chloride hexahydrate and 300 mM acetate buffer (pH 3.6) at 1:1:10 (*v*/*v*/*v*). The freshly prepared FRAP reagent (200 μL) was added to solutions of tested compounds at different concentrations (20 μL) and thoroughly mixed in a 96-well microplate. The absorbance of a blue ferrous tripyridyltriazine complex (Fe^2+^/TPTZ) was read after 4 min of incubation in the dark at 593 nm using a Multiskan GO microplate spectrophotometer (Thermo Fisher Scientific; Waltham, MA, USA). Results were expressed in Fe^2+^ μM. All measurements were performed in triplicate in the concentration range 5–1000 μM of the analyzed compounds.

### 3.4. Direct Methylglyoxal and Glyoxal Trapping Capacity

Methylglyoxal and glyoxal direct trapping capacity was investigated according to the slightly modified Sang et al. [71] method. Briefly, 0.6 mM methylglyoxal (MGO) or glyoxal (GO) was incubated for 1 h with 0.2 mM of rutin, troxerutin, diosmin, diosmetin, hesperidin, hesperetin, calcium dobesilate, metformin hydrochloride, and quercetin in 100 mM phosphate buffer saline (PBS; pH 7.4) at 37 °C to equal physiological conditions and shaken at 40 revolutions per minute. The incubation reaction was terminated by adding 2.5 μL of acetic acid (≥99%) and placing the collected samples in an ice water bath. The samples were further filtered through hydrophilic Millex Syringe Filters (Durapore 0.22 μm; Millipore, Burlington, MA, USA) and analyzed using UHPLC-ESI-MS to investigate their ability to form adducts with methylglyoxal and glyoxal. The trapping agent solutions were freshly prepared before each series of experiments was begun, and the pH of the sodium phosphate buffer was determined immediately before use.

### 3.5. Time Course Study of MGO and GO Scavenging Reaction

The time course of the MGO and GO scavenging reaction was investigated for the compounds’ indicated activity in methylglyoxal and glyoxal direct quenching assay in UHPLC-ESI-QqTOF-MS analysis. The study was carried out according to the method of Shao et al. [62] with slight modification. The direct trapping study found using an excess of MGO and GO allows compounds to form both mono- and di-adducts with α-oxoaldehydes. Therefore, methylglyoxal or glyoxal at a final concentration of 0.6 mM and each selected compound at 0.2 mM were incubated in 100 mM phosphate buffer saline (PBS; pH 7.4) at 37 °C with a speed of 40 revolutions per minute to simulate physiological conditions for 1, 2, 4, 8, and 24 h. Afterward, 500 μL of reaction mixtures were collected at each time point and placed in an ice water bath, then 2.5 μL of acetic acid (≥99%) was added to stop the reaction. The derivatization of the remaining MGO and GO in an aliquot amount of each sample was carried out by adding 1,2-phenylenediamine (PDA) at 100 mM and shaken by vortex for 5 s. The mixtures were kept at ambient temperature for 30 min for derivatization of the remaining methylglyoxal or glyoxal to complete. The UHPLC analysis was used to measure the amount of methylquinoxaline and quinoxaline formed through the reaction of methylglyoxal and glyoxal with PDA, respectively. The stock solutions of MGO, GO, and PDA were prepared immediately before the beginning of the study. The pH of the phosphate buffer was also determined shortly before the experiment.

### 3.6. UHPLC-ESI-QqTOF-MS Analysis

The study of the formation of methylglyoxal and glyoxal adducts was performed using the same UHPLC system configured as above (Section 4), interfaced with Compact ESI-QqTOF-MS (Bruker Daltonics; Bremen, Germany). Mobile phases consisted of A (0.1% formic acid in water) and B (0.1% acetic acid in 100% acetonitrile). The following gradient mobile phase program at a flow rate of 0.3 mL/min was used: 0–12 min, 97–65% A in B; 12–14 min, 65% A in B; 14–17 min, 65–20% A in B; 17–19 min 20% A in B. Then, the system returned to the initial setting and washed with 97% A in B until the system was stabilized before the next analysis. Both positive and negative ion modes were used for data acquisition. As a nebulizing and drying gas, nitrogen was used at temperature 210 °C, 2.0 bar pressure, and flow 0.8 L/min. For internal calibration sodium formate clusters (10 mM) were used. The injection volume was 1 mL. Additional operating conditions of the mass spectrometer were as follows: capillary voltage was set at 5 kV (ESI^+^, ESI^−^), collisional energy was 8.0 eV, and for the MS/MS mode, it was 35 and 40 eV. The data acquisition and processing were carried out with Compass Data Analysis software (Bruker Daltonics; Bremen, Germany.

### 3.7. UHPLC-DAD Analysis

Derivatized methylglyoxal and glyoxal were analyzed by the Thermo Scientific Dionex UltiMate 3000 UHPLC system (Thermo Fisher Scientific; Waltham, MA, USA) incorporated with a quaternary pump (LPG-3400D), UltiMate 3000 RS autosampler (WPS-3000), and fast separation photodiode array detector (DAD-3000). Derivatives were separated on a Kinetex C18 column (150 × 2.1 mm × 2.6 μm) (Phenomenex; Torrance, CA, USA) and a temperature-controlled column compartment (TCC–3000) was used to maintain its temperature at 40 °C. The binary mobile phase system consisted of 0.1% (*v*/*v*) formic acid in water (solvent C) and 0.1% (*v*/*v*) formic acid in acetonitrile (solvent D). The column eluted with a binary gradient system at a flow rate of 400 μL/min: 100% C from 0 to 4 min, 100–77% C in D from 4 to 25 min, and held at 77% C in D for 0.5 min, 77–100% C in D from 25.5 to 30 min, and then 100% C from 30 to 32 min. The injection volume was 10 μL. The peak areas of methylquinoxaline and quinoxaline were monitored at wavelengths of 316 and 314 nm, respectively. Data acquisition was carried out with the Chromeleon Chromatography Data System (Thermo Fisher Scientific; Waltham, MA, USA).

### 3.8. Linearity and Calibration of UHPLC-DAD Method

The applied UHPLC-DAD method was validated by the determination of linearity, LOD, and LOQ. Calibration equations for quantified metylquinoxaline and quinoxaline were assessed at seven concentration levels, and triplicate injections were performed for each concentration. The values of LOD were established at a signal-to-noise ratio (S/N) of 0.3 and LOQ was calculated at S/N of 1. Results of the method validation for standards are shown in Table 4.

### 3.9. Statistical Analysis

Data were analyzed using the Shapiro–Wilk test to assess normality of distribution, followed by one-way analysis of variance (ANOVA) with Tukey’s multiple comparison test using the GraphPad Prism 9 software. *p* values equal or less than 0.05 were considered significant.

## 4. Conclusions

To conclude, in addition to other therapeutic applications, some vasoprotective medicines and their structural analogs such as hesperetin, diosmetin, quercetin, and calcium dobesilate could be considered as potential antiglycative agents against glycation-related complications of diabetes. While the current study has clearly indicated that tested VPs and their structural analogs can effectively trap MGO and GO or reduce RCS-mediated glycation, a key limitation that must be considered is that the results presented here are only from in vitro models. The relevance of these results to humans must be experimentally proven using in vivo models. Although RCS trapping and reducing are simple, direct chemical reactions, which may suggest that the in vitro results have a high probability of being confirmed in vivo, the bioavailability of individual substances should also be taken into account.

## Figures and Tables

**Table 1:** Antioxidant activity of selected vasoprotectives and their structural analogs compared to metformin.

Sample	FRAP	ABTS	
	Fe(II) ^a^ [μM]	IC50 [µM]	Inhibition [%]
Rutin	30.14 ± 0.20	2.41 ± 0.05	69.17 ± 0.24 ^b^
Quercetin	133.29 ± 0.30	3.81 ± 0.04	66.40 ± 1.12 ^b^
Troxerutin	1.68 ± 0.43	24.12 ± 2.42	17.63 ± 0.93 ^b^
Diosmin	5.77 ± 0.20	11.10 ± 1.44	30.41 ± 1.86 ^b^
Diosmetin	25.62 ± 0.41	7.05 ± 0.05	39.92 ± 0.26 ^b^
Hesperidin	53.08 ± 0.51	5.21 ± 0.07	48.94 ± 0.57 ^b^
Hesperetin	99.27 ± 0.92	5.67 ± 0.13	49.80 ± 0.73 ^b^
Calcium dobesilate	54.98 ± 0.65	5.13 ± 0.19	51.35 ± 0.88 ^b^
Metformin	n.a.	n.a.	n.a.
Trolox	n.t.	11.29 ± 0.91	29.04 ± 0.81 ^c^

Values are mean triplicate (*n* = 3); ^a^ calculated for samples at final concentration ~4.9 µM; ^b^ calculated for samples at final concentration ~9.1 µM; ^c^ calculated for samples at final concentration ~7.9 µM; n.a., no activity; n.t., not tested; FRAP assay antioxidant activity values were expressed as Fe^2+^ iron ion equivalent; ABTS assay antioxidant activity values were expressed as percent inhibition and concentration required for a 50% reduction of radical activity.

**Table 2:** Adducts of methylglyoxal and investigated compounds formed after 1 h of incubation in pH 7.4 of phosphate buffer solution at 37 °C.

Compound	Peak	Rt [min]	[M − H]^−^ or [M + H]^+^ Mono-MGO Adduct (*m*/*z*)	MS/MS (*m*/*z*)	Peak	Rt [min]	[M − H]^–^ or [M + H]^+^ Di-MGO Adduct (*m*/*z*)	MS/MS (*m*/*z*)
Rutin ^1^	a	8.69	681.1698	663, 609, 301	a	7.90	753.1885	681, 644, 609, 301
	b	8.84	681.1701	663, 609, 301	b	8.26	753.1890	681, 644, 609, 301
	c	8.85	681.1684	663, 609, 301	c	8.44	753.1840	681, 644, 609, 301
Quercetin ^2^	a	10.93	373.0567	n.d.	a	10.01	445.0779	n.d.
	b	11.06	373.0562					
Troxerutin	-	-	n.d.	n.d.	-	-	n.d.	n.d.
Diosmin	-	-	n.d.	n.d.	-	-	n.d.	n.d.
Diosmetin ^3^	a	12.19	371.0758	353, 338, 299	a	11.24	443.0970	425, 410, 299
	b	12.36	371.0766	353, 338, 299				
Hesperidin ^4^	a	8.97	681.1972	609, 373, 301	-	-	n.d.	n.d.
	b	9.19	681.1981	609, 373, 301				
	c	9.82	681.1966	609, 373, 301				
	d	9.95	681.1979	609, 373, 301				
	e	10.88	681.1965	609, 373, 301				
	f	10.98	681.1960	609, 373, 301				
Hesperetin ^5^	a	12.04	373.0913	355, 340, 301	a	10.80	445.1103	427, 409
	b	12.93	373.0899	355, 340, 301				
Calcium dobesilate	-	-	n.d.	n.d.	-	-	n.d.	n.d.
Metformin ^6^	a	1.48	184.1158	n.d.	a	1.57	256.1297	n.d.
	b	1.20	202.1229		b	1.64	256.1300	

Pseudomolecular ion (*m*/*z*): ^1^ 609; ^2^ 301; ^3^ 299; ^4^ 609; ^5^ 301; ^6^ 130; Rt, retention time; n.d., not detected; letters of the alphabet (a–f) represent different isomers of the same compound.

**Table 3:** Adducts of glyoxal and investigated compounds formed after 1 h of incubation in pH 7.4 phosphate buffer solution at 37 °C.

Compound	Peak	Rt [min]	[M − H]^−^ or [M + H]^+^ Mono-GO Adduct (*m*/*z*)	MS/MS (*m*/*z*)	Peak	Rt [min]	[M − H]^−^ or [M + H]^+^ Di-GO Adduct (*m*/*z*)	MS/MS (*m*/*z*)
Rutin ^1^	a	8.09	667.1445	n.d.	-	-	n.d.	n.d.
	b	8.41	667.1448	n.d.				
Quercetin ^2^	-	-	n.d.	n.d.	-	-	n.d.	n.d.
Troxerutin	-	-	n.d.	n.d.	-	-	n.d.	n.d.
Diosmin	-	-	n.d.	n.d.	-	-	n.d.	n.d.
Diosmetin ^3^	a	11.64	357.0605	324, 313, 299, 284	-	-	n.d.	n.d.
	b	12.1	357.0602	324, 313, 299, 284				
Hesperidin ^4^	a	11.05	677.1861	609, 301	-	-	n.d.	n.d.
	b	11.37	677.1844	609, 301				
Hesperetin ^5^	a	11.28	359.0771	341, 326, 301	a	12.99	417.0808	399, 301
	b	12.37	359.0762	341, 326, 301				
	c	13.78	359.0768	341, 326, 301				
Calcium dobesilate	-	-	n.d.	n.d.	-	-	n.d.	n.d.
Metformin	-	-	n.d.	n.d.	-	-	n.d.	n.d.

Pseudomolecular ion (*m*/*z*): ^1^ 609; ^2^ 301; ^3^ 299; ^4^ 609; ^5^ 301; Rt, retention time; n.d., not detected; letters of the alphabet (a–c) represent different isomers of the same compound.

**Table 4:** Validation parameters of the UHPLC-DAD method.

Compound	Method	λ [nm]	Linear Equation	R^2^	Range [µM]	LOD [µM]	LOQ [µM]
Methyloquinoxaline	UHPLC-DAD	316	y = 2739.8843x + 5.0076	0.9995	5–210	0.29	0.96
Quinoxaline	UHPLC-DAD	314	y = 325.4515x + 1.7417	0.9998	5–210	0.19	0.64

λ, wavelength; y = ax + b; y, peak area; R^2^, coefficient of determination; LOD, limit of detection; LOQ, limit of quantitation; *n* = 3 × 7.

## Data Availability

Data supporting reported results are available from the corresponding author.

## References

[1] Avogaro A., Albiero M., Menegazzo L., De Kreutzenberg S., Fadini G.P. (2011). Endothelial dysfunction in diabetes: The role of reparatory mechanisms. Diabetes Care.

[2] Janda K., Krzanowski M., Gajda M., Dumnicka P., Jasek E., Fedak D., Pietrzycka A., Kuźniewski M., Litwin J.A., Sułowicz W. (2015). Vascular effects of advanced glycation end-products: Content of immunohistochemically detected AGEs in radial artery samples as a predictor for arterial calcification and cardiovascular risk in asymptomatic patients with chronic kidney disease. Dis. Markers.

[3] Grundy S.M., Benjamin I.J., Burke G.L., Chait A., Eckel R.H., Howard B.V., Mitch W., Smith S.C., Sowers J.R. (1999). Diabetes and cardiovascular disease: A statement for healthcare professionals from the american heart association. Circulation.

[4] Lunder M., Janić M., Šabovič M. (2019). Prevention of Vascular Complications in Diabetes Mellitus Patients: Focus on the Arterial Wall. Curr. Vasc. Pharmacol..

[5] Chaudhuri J., Bains Y., Guha S., Kahn A., Hall D., Bose N., Gugliucci A., Kapahi P. (2018). The Role of Advanced Glycation End Products in Aging and Metabolic Diseases: Bridging Association and Causality. Cell Metab..

[6] Salahuddin P., Rabbani G., Khan R.H. (2014). The role of advanced glycation end products in various types of neurodegenerative disease: A therapeutic approach. Cell. Mol. Biol. Lett..

[7] Odani H., Shinzato T., Matsumoto Y., Usami J., Maeda K. (1999). Increase in three α,β-dicarbonyl compound levels in human uremic plasma: Specific in vivo determination of intermediates in advanced Maillard reaction. Biochem. Biophys. Res. Commun..

[8] Murakami M., Simons M. (2009). Regulation of vascular integrity. J. Mol. Med..

[9] Liakopoulos V., Roumeliotis S., Gorny X., Eleftheriadis T., Mertens P.R. (2017). Oxidative stress in patients undergoing peritoneal dialysis: A current review of the literature. Oxid. Med. Cell. Longev..

[10] Semchyshyn H.M. (2014). Reactive carbonyl species in vivo: Generation and dual biological effects. Sci. World J..

[11] Mukohda M., Yamawaki H., Nomura H., Okada M., Hara Y. (2009). Methylglyoxal inhibits smooth muscle contraction in isolated blood vessels. J. Pharmacol. Sci..

[12] Miyata T., Sugiyama S., Saito A., Kurokawa K. (2001). Reactive carbonyl compounds related uremic toxicity. Kidney Int. Suppl..

[13] Rodrigues T., Matafome P., Santos-Silva D., Sena C., Seiça R. (2013). Reduction of methylglyoxal-induced glycation by pyridoxamine improves adipose tissue microvascular lesions. J. Diabetes Res..

[14] Iacobini C., Vitale M., Pesce C., Pugliese G., Menini S. (2021). Diabetic complications and oxidative stress: A 20-year voyage back in time and back to the future. Antioxidants.

[15] Borg D.J., Forbes J.M. (2016). Targeting advanced glycation with pharmaceutical agents: Where are we now?. Glycoconj. J..

[16] Li X., Zheng T., Sang S., Lv L. (2014). Quercetin inhibits advanced glycation end product formation by trapping methylglyoxal and glyoxal. J. Agric. Food Chem..

[17] Han L., Lin Q., Liu G., Han D., Niu L., Su D. (2019). Catechin inhibits glycated phosphatidylethanolamine formation by trapping dicarbonyl compounds and forming quinone. Food Funct..

[18] Shin E.R., Jung W., Kim M.K., Chong Y. (2018). Identification of (−)-epigallocatechin (EGC) as a methylglyoxal (MGO)-trapping agent and thereby as an inhibitor of advanced glycation end product (AGE) formation. Appl. Biol. Chem..

[19] Wang P., Chen H., Sang S. (2016). Trapping Methylglyoxal by Genistein and Its Metabolites in Mice. Chem. Res. Toxicol..

[20] Shao X. (2010). Scavenging Effects of Dietary Flavonoids on Reactive Dicaronyl Species and Their Possible Implications on the Inhibition of the Formation of Advanced Glycation-End Products. Ph.D. Thesis.

[21] Zhang S., Xiao L., Lv L., Sang S. (2020). Trapping methylglyoxal by myricetin and its metabolites in mice. J. Agric. Food Chem..

[22] Kinsky O.R., Hargraves T.L., Anumol T., Jacobsen N.E., Dai J., Snyder S.A., Monks T.J., Lau S.S. (2016). Metformin Scavenges Methylglyoxal to Form a Novel Imidazolinone Metabolite in Humans. Chem. Res. Toxicol..

[23] Gohel M., Davies A. (2009). Pharmacological Agents in the Treatment of Venous Disease: An Update of the Available Evidence. Curr. Vasc. Pharmacol..

[24] Rees A., Dodd G.F., Spencer J.P.E. (2018). The effects of flavonoids on cardiovascular health: A review of human intervention trials and implications for cerebrovascular function. Nutrients.

[25] Cium L., Milaciu M.V., Runcan O., Vesa C., Negrean V., Pern M., Donca V.I. (2020). The Effects of Flavonoids in Cardiovascular Diseases. Molecules.

[26] Ullah A., Munir S., Badshah S.L., Khan N., Ghani L., Poulson B.G., Emwas A., Jaremko M. (2020). Important Flavonoids and Their Role as a Therapeutic Agent. Molecules.

[27] Lemmens-Gruber R., Marchart E., Rawnduzi P., Engel N., Benedek B., Kopp B. (2006). Investigation of the spasmolytic activity of the flavonoid fraction of Achillea millefolium s.l. on isolated guinea-pig ilea. Arzneim.-Forsch./Drug Res..

[28] Kim M.H. (2003). Flavonoids inhibit VEGF/bFGF-induced angiogenesis in vitro by inhibiting the matrix-degrading proteases. J. Cell. Biochem..

[29] Jakimiuk K., Gesek J., Atanasov A.G., Tomczyk M. (2021). Flavonoids as inhibitors of human neutrophil elastase. J. Enzyme Inhib. Med. Chem..

[30] Maessen D.E.M., Stehouwer C.D.A., Schalkwijk C.G. (2015). The role of methylglyoxal and the glyoxalase system in diabetes and other age-related diseases. Clin. Sci..

[31] Nenna A., Nappi F., Avtaar Singh S., Sutherland F., Di Domenico F., Chello M., Spadaccio C. (2015). Pharmacologic approaches against Advanced Glycation End Products (AGEs) in diabetic cardiovascular disease. Res. Cardiovasc. Med..

[32] Bolton W.K., Cattran D.C., Williams M.E., Adler S.G., Appel G.B., Cartwright K., Foiles P.G., Freedman B.I., Raskin P., Ratner R.E. (2004). Randomized Trial of an Inhibitor of Formation of Advanced Glycation End Products in Diabetic Nephropathy. Am. J. Nephrol..

[33] Kiho T., Kato M., Usui S., Hirano K. (2005). Effect of buformin and metformin on formation of advanced glycation end products by methylglyoxal. Clin. Chim. Acta.

[34] Beisswenger P.J., Ruggiero-Lopez D. (2003). Metformin inhibition of glycation processes. Diabetes Metab..

[35] Li D., Mitsuhashi S., Ubukata M. (2012). Protective effects of hesperidin derivatives and their stereoisomers against advanced glycation end-products formation. Pharm. Biol..

[36] Matsuda H., Wang T., Managi H., Yoshikawa M. (2003). Structural requirements of flavonoids for inhibition of protein glycation and radical scavenging activities. Bioorg. Med. Chem..

[37] Liu J., Li S., Sun D. (2019). Calcium Dobesilate and Micro-vascular diseases. Life Sci..

[38] Vojnicovic B. (1991). Doxium (Calcium dobesilate) reducesblood hyperviscosity and lowers elevated intraocular pressure in patients with diabetic rethinopathy and glaucoma. Ophthalmic Res..

[39] Mehta R., Wong L., O’Brien P.J. (2009). Cytoprotective mechanisms of carbonyl scavenging drugs in isolated rat hepatocytes. Chem. Biol. Interact..

[40] Sena C.M., Matafome P., Crisóstomo J., Rodrigues L., Fernandes R., Pereira P., Seiça R.M. (2012). Methylglyoxal promotes oxidative stress and endothelial dysfunction. Pharmacol. Res..

[41] Reddy V.P., Beyaz A. (2006). Inhibitors of the Maillard reaction and AGE breakers as therapeutics for multiple diseases. Drug Discov. Today.

[42] Tiveron A.P., Melo P.S., Bergamaschi K.B., Vieira T.M.F.S., Regitano-D’Arce M.A.B., Alencar S.M. (2012). Antioxidant activity of Brazilian vegetables and its relation with phenolic composition. Int. J. Mol. Sci..

[43] Giuffrè A.M. (2019). Bergamot (*Citrus bergamia*, Risso): The effects of cultivar and harvest date on functional properties of juice and cloudy juice. Antioxidants.

[44] Payne A.C., Mazzer A., Clarkson G.J.J., Taylor G. (2013). Antioxidant assays—Consistent findings from FRAP and ORAC reveal a negative impact of organic cultivation on antioxidant potential in spinach but not watercress or rocket leaves. Food Sci. Nutr..

[45] Ilyasov I.R., Beloborodov V.L., Selivanova I.A., Terekhov R.P. (2020). ABTS/PP decolorization assay of antioxidant capacity reaction pathways. Int. J. Mol. Sci..

[46] Plumb G.W., Price K.R., Williamson G. (1999). Antioxidant properties of flavonol glycosides from tea. Redox Rep..

[47] Ouslimani N., Peynet J., Bonnefont-Rousselot D., Thérond P., Legrand A., Beaudeux J.L. (2005). Metformin decreases intracellular production of reactive oxygen species in aortic endothelial cells. Metabolism.

[48] Logie L., Harthill J., Patel K., Bacon S., Hamilton D.L., Macrae K., McDougall G., Wang H.H., Xue L., Jiang H. (2012). Cellular responses to the metal-binding properties of metformin. Diabetes.

[49] Hosseinimehr S.J., Ghasemi F., Flahatgar F., Rahmanian N., Ghasemi A., Asgarian-Omran H. (2020). Atorvastatin sensitizes breast and lung cancer cells to ionizing radiation. Iran. J. Pharm. Res..

[50] Dadpisheh S., Ahmadvand H., Jafaripour L., Nouryazdan N., Babaeenezhad E., Shati H., Bagheri S. (2020). Effect of troxerutin on oxidative stress induced by sciatic nerve ischemia-reperfusion injury in rats. J. Kerman Univ. Med. Sci..

[51] Badarinath A.V., Rao K.M., Madhu C., Chetty S., Ramkanth S., Rajan T.V.S., Gnanaprakash K. (2010). A Review on in-vitro antioxidant methods: Comparisions, correlations and considerations. Int. J. PharmTech Res..

[52] Zhang X.Y., Liu W., Wu S.S., Jin J.L., Li W.H., Wang N.L. (2015). Calcium dobesilate for diabetic retinopathy: A systematic review and meta-analysis. Sci. China Life Sci..

[53] Hollman P.C.H., Van Trijp J.M.P., Buysman M.N.C.P., Martijn M.S., Mengelers M.J.B., De Vries J.H.M., Katan M.B. (1997). Relative bioavailability of the antioxidant flavonoid quercetin from various foods in man. FEBS Lett..

[54] Jin F., Nieman D.C., Shanely R.A., Knab A.M., Austin M.D., Sha W. (2010). The variable plasma quercetin response to 12-week quercetin supplementation in humans. Eur. J. Clin. Nutr..

[55] Kanaze F.I., Bounartzi M.I., Georgarakis M., Niopas I. (2007). Pharmacokinetics of the citrus flavanone aglycones hesperetin and naringenin after single oral administration in human subjects. Eur. J. Clin. Nutr..

[56] Guo X., Hou L., Yin Y., Wu J., Zhao F., Xia L., Cheng X., Liu Q., Liu L., Xu E. (2018). Negative interferences by calcium dobesilate in the detection of five serum analytes involving Trinder reaction-based assays. PLoS ONE.

[57] Lo C.Y., Hsiao W.T., Chen X.Y. (2011). Efficiency of trapping methylglyoxal by phenols and phenolic acids. J. Food Sci..

[58] Yoon S.R., Shim S.M. (2015). Inhibitory effect of polyphenols in Houttuynia cordata on advanced glycation end-products (AGEs) by trapping methylglyoxal. LWT-Food Sci. Technol..

[59] Bednarska K., Kuś P., Fecka I. (2020). Investigation of the phytochemical composition, antioxidant activity, and methylglyoxal trapping effect of *Galega officinalis* L. Herb in vitro. Molecules.

[60] Wang W., Qi Y., Rocca J.R., Sarnoski P.J., Jia A., Gu L. (2015). Scavenging of Toxic Acrolein by Resveratrol and Hesperetin and Identification of Adducts. J. Agric. Food Chem..

[61] Nemet I., Varga-Defterdarović L., Turk Z. (2004). Preparation and quantification of methylglyoxal in human plasma using reverse-phase high-performance liquid chromatography. Clin. Biochem..

[62] Shao X., Chen H., Zhu Y., Sedighi R., Ho C.T., Sang S. (2014). Essential structural requirements and additive effects for flavonoids to scavenge methylglyoxal. J. Agric. Food Chem..

[63] Zhu H., Poojary M.M., Andersen M.L., Lund M.N. (2020). The effect of molecular structure of polyphenols on the kinetics of the trapping reactions with methylglyoxal. Food Chem..

[64] Van Den Eynde M.D.G., Geleijnse J.M., Scheijen J.L.J.M., Hanssen N.M.J., Dower J.I., Afman L.A., Stehouwer C.D.A., Hollman P.C.H., Schalkwijk C.G. (2018). Quercetin, but not epicatechin, decreases plasma concentrations of methylglyoxal in adults in a randomized, double-blind, placebo-controlled, crossover trial with pure flavonoids. J. Nutr..

[65] Walle T. (2004). Absorption and metabolism of flavonoids. Free Radic. Biol. Med..

[66] Murota K., Nakamura Y., Uehara M. (2018). Flavonoid metabolism: The interaction of metabolites and gut microbiota. Biosci. Biotechnol. Biochem..

[67] Boersma M.G., Van der Woude H., Bogaards J., Boeren S., Vervoort J., Cnubben N.H.P., Van Iersel M.L.P.S., Van Bladeren P.J., Rietjens I.M.C.M. (2002). Regioselectivity of phase II metabolism of luteolin and quercetin by UDP-glucuronosyl transferases. Chem. Res. Toxicol..

[68] Liu W., Ma H., Frost L., Yuan T., Dain J.A., Seeram N.P. (2014). Pomegranate phenolics inhibit formation of advanced glycation endproducts by scavenging reactive carbonyl species. Food Funct..

[69] Chen L., Kang Y.H. (2014). Antioxidant and enzyme inhibitory activities of plebeian herba (*Salvia plebeia* R. Br.) under different cultivation conditions. J. Agric. Food Chem..

[70] Blois M.S. (1958). Antioxidant determinations by the use of a stable free radical. Nature.

[71] Sang S., Shao X., Bai N., Lo C.Y., Yang C.S., Ho C.T. (2007). Tea polyphenol (−)-epigallocatechin-3-gallate: A new trapping agent of reactive dicarbonyl species. Chem. Res. Toxicol..

